# Annexin A1 Mimetic Peptide Ac_2-26_ Modulates the Function of Murine Colonic and Human Mast Cells

**DOI:** 10.3389/fimmu.2021.689484

**Published:** 2021-09-07

**Authors:** Marcia Pereira Oliveira, Janesly Prates, Alexandre Dantas Gimenes, Silvia Graciela Correa, Sonia Maria Oliani

**Affiliations:** ^1^Laboratory of Interdisciplinary Medical Research, Oswaldo Cruz Institute, Fiocruz, Rio de Janeiro, Brazil; ^2^Department of Biology, Institute of Bioscience, Humanities and Exact Science, São Paulo State University (Unesp), São José do Rio Preto, Brazil; ^3^Department of Functional Morphology, Faceres School of Medicine, São José do Rio Preto, Brazil; ^4^Departamento de Bioquímica Clinica-Centro de Investigaciones en Bioquímica Clínica e Inmunología (CIBICI - CONICET) - Facultad de Ciencias Quimicas- Universidad Nacional de Córdoba (UNC), Córdoba, Argentina; ^5^Advanced Research Center in Medicine, CEPAM –Unilago, São José do Rio Preto, Brazil; ^6^Federal University of São Paulo, Post Graduate Program in Structural and Functional Biology, Escola Paulista de Medicina (Unifesp-EPM), São Paulo, Brazil

**Keywords:** mast cell (MC), compound 48/80, human MC line (HMC-1), colon explant culture, annexin A1, IL-4, FPRs

## Abstract

Mast cells (MCs) are main effector cells in allergic inflammation and after activation, they release stored (histamine, heparin, proteases) and newly synthesized (lipid mediators and cytokines) substances. In the gastrointestinal tract the largest MC population is located in the lamina propria and submucosa whereas several signals such as the cytokine IL-4, seem to increase the granule content and to stimulate a remarkable expansion of intestinal MCs. The broad range of MC-derived bioactive molecules may explain their involvement in many different allergic disorders of the gastrointestinal tract. Annexin A1 (AnxA1) is a 37 KDa glucocorticoid induced monomeric protein selectively distributed in certain tissues. Its activity can be reproduced by mimetic peptides of the N-terminal portion, such as Ac_2-26_, that share the same receptor FPR-L1. Although previous reports demonstrated that AnxA1 inhibits MC degranulation in murine models, the effects of exogenous peptide Ac_2-26_ on intestinal MCs or the biological functions of the Ac_2-26_/FPR2 system in human MCs have been poorly studied. To determine the effects of Ac_2-26_ on the function of MCs toward the possibility of AnxA1-based therapeutics, we treated WT and IL-4 knockout mice with peptide Ac2-26, and we examined the spontaneous and compound 48/80 stimulated colonic MC degranulation and cytokine production. Moreover, *in vitro*, using human mast cell line HMC-1 we demonstrated that exogenous AnxA1 peptide is capable of interfering with the HMC-1 degranulation in a direct pathway through formyl peptide receptors (FPRs). We envisage that our results can provide therapeutic strategies to reduce the release of MC mediators in inflammatory allergic processes.

## Introduction

Mast cells (MCs) have been identified as the main effector cells for allergic inflammation. They are distributed in specific sites such as skin, blood vessels, respiratory and intestinal mucosa, contributing to host defenses. The classical and most effective mechanism for MC activation is cross-linking of cell-surface bound IgE to its high-affinity receptor (FcϵRI) by allergen in acute allergic reactions. After activation, MCs are able to release newly synthesized (lipid mediators and cytokines) and stored (histamine, heparin, proteases) substances which are contained in their cytoplasmic lipid bodies and granules (1–3). In mice, the ability of MCs to respond to particular stimuli, can be modulated by cytokines, growth factors, and other microenvironmental signals. The heterogeneity of MC subpopulations depends on the anatomical site in which they reside, allowing improved flexibility and diversity of responsiveness (4). Considering that MCs complete the differentiation and maturation in target tissues in the presence of local trophic factors such as IL-9, IL-10, IL-3, IL-4, IL-33, CXCL12 (5), the absence of some of these signals could determine differences on the biological activity of these cells.

The normal gastrointestinal tract (GI) contains the largest MC population in the lamina propria of the mucosa and in the submucosa. This amount can increase up to tenfold in the course of many intestinal diseases. Several findings support that the function of MCs in the GI is not limited to the antigen-reactive effector cells, playing a central role in the control of the barrier fitness and the transport properties of the intestinal epithelium (6). Mucosal MCs can respond to both IgE/antigen-dependent and non-IgE-dependent stimulation by a mechanism depending on the release of bioactive mediators into adjacent tissues that leads to physiological responses (7). Recently, it became clear that MC regulation is essential for normal functioning of the bowel tissues (8).

The broad range of MC-derived bioactive molecules may explain their involvement in many different pathological conditions including infections, neoplastic diseases as well as many types of allergic disorders of the GI (9). Studies in hypersensitivity and stress models have shown that alterations in mucosal function are attributable to either direct action on epithelial receptors by MC mediators and/or indirect action by neurotransmitters (10). IL-4 is well known as a Th2 cell differentiation promoter although some role in directing Th1 responses has been claimed (11). Cells of several lineages produce IL-4, including CD4 and CD8 T cells, NKT cells, eosinophils, MCs and basophils. IL-4 has been shown not only to elicit the FcϵRI expression but also to increase the granule content (12). While several reports found normal/similar numbers of MCs in IL-4-deficient mice other authors demonstrated that the cytokine stimulates a remarkable expansion of intestinal MCs from enhanced signaling through the IL-4Rα chain in rodent model of food allergy (13). Remarkably, differences in the effects of the compound 48/80 have been detected studying humoral immunity in WT mice and MC deficient mice (14).

Annexin A1 (AnxA1) is a 37 KDa glucocorticoid induced monomeric protein with selective distribution in certain tissues. It is synthetized in some immune cells, mainly myeloid cells including macrophages, MCs, eosinophils, and neutrophils, beyond the neuroendocrine system (15–17). Glucocorticoids not only stimulate AnxA1 transcription, but also induce the release into the cytoplasm of its pre-existing forms *via* a receptor-dependent, non-genomic pathway, preceded by phosphorylation at key sites in the N-terminus and other sites (18). The activity of this protein can be reproduced by mimetic peptides of the N-terminal portion (19) including Ac_2-26_, that have been investigated in many acute (20, 21), chronic (17, 22) and systemic (23) inflammation models. AnxA1 and its active derived peptide Ac_2-26_ share the same receptor FPR-L1 (FPR2/ALX in man) which inhibits cell activation in an autocrine or paracrine pathway (24).

AnxA1 induces the expression of IL-4 and IL-10 in inflammatory conditions (25), two cytokines that play an important role in the regulation of peripheral intestinal tolerance (26, 27). Also, pro-inflammatory cytokines such as IL-6 and TNF-α may induced the AnxA1 protein expression (28). Our research group showed that the induction of peritonitis promotes both reorganization of cytoplasmic granules and *de novo* synthesis of AnxA1, with important effects in the regulation of the inflammatory infiltrate and cytokine production in the mesentery (16).

We have reported the localization of AnxA1 in connective tissue MCs, and that its expression is susceptible not only to glucocorticoid treatment, but also to acute inflammatory response (29). Moreover, previous study demonstrated that AnxA1 inhibits MC degranulation in rodent models (30). However, relatively little is known regarding on the effects of AnxA1 on the biological functions of human MCs and more studies are required to comprehend the effects of exogenous AnxA1/FPR2 system.

While cell lines derived from tumor tissues differ from normal primary MCs, valuable information can be obtained from the HMC-1 cell line derived from a MC leukaemia patient. Although HMC-1 cells are immature, they display many primary characteristics of tissue MCs, such as histamine, heparin, β-hexosaminidase and tryptase expression (31–33). Based on these data, HMC-1 cells are suitable platform for the study of human MC degranulation in acute response (34).

Building upon these observations, we considered the possibility that the effect of Ac_2-26_ peptide could be specific in WT and IL-4 knockout mice. Considering that type 2 responses are implicated in several aspects of gut homeostasis but also in chronic intestinal inflammation, we examined the spontaneous and acute stimulated colonic MC degranulation and cytokine production under Ac_2-26_ treatment in both mouse strains. Moreover, using the HMC-1 cell line we demonstrated that exogenous AnxA1 peptide is capable to interfere with MC degranulation triggered by acute stimulation through formyl peptide receptors (FPRs).

## Material and Methods

### Animals

All animal experiments were performed in accordance with the Argentina animal welfare legislation and approved by the Committee on the Ethics of Animal Experiments from Cordoba National University (resolution 1412/2012). Six- to eight-week-old male C57BL/6 wild type (WT) and C57BL/6 interleukin-4- knockout (IL-4 KO) mice maintained under specific-pathogen-free conditions were used for all experiments. The animals were housed with a 12-h light–dark cycle and were allowed food and water ad libitum.

### Treatment Protocol

**Figure 1:**
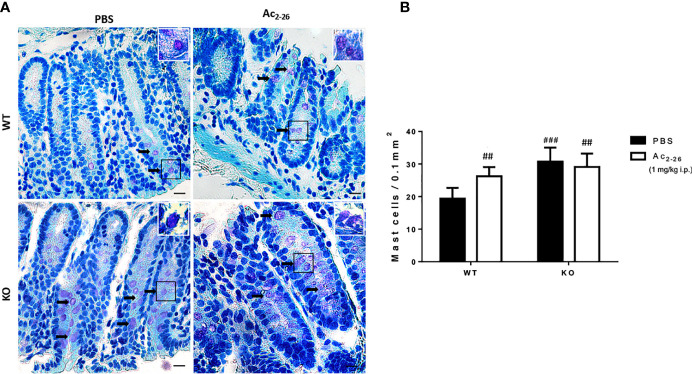
Histological analysis of the distal colon of C57BL/6 WT and C57BL/6 IL-4 KO mice. **(A)** Representative colon sections after PBS or peptide Ac2-26 treatment (1 mg/kg i.p.). Arrows indicate examples of MCs in the tissue. Insets show higher magnification. Staining: Toluidine Blue. Sections: 4μm. Scale bars: 20 μm. **(B)** Quantitative analysis of MCs in the colon fragments. Experiments were performed twice with n = 4 animals/group. Results of cell numbers/mm^2^ are expressed as the mean ± S.D, n = 8. Data were analyzed using one-way ANOVA. ^##^p <0.01 compared to WT PBS group. ^###^p <0.001 compared to WT PBS group.

**Figure 2:**
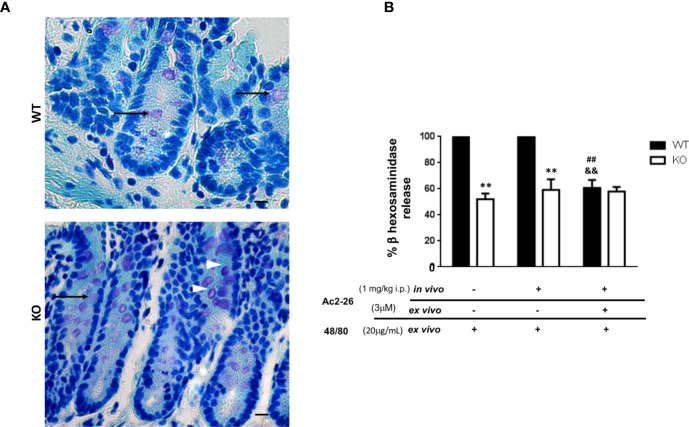
Effect of peptide Ac2-26 on MC degranulation in colon explants. C57BL/6 WT and C57BL/6 interleukin-4- KO mice were pre-treated with PBS (-) or 3µM Ac2 -26 (+) and stimulated with 20µg/mL compound 48/80 *ex vivo* for 24 (h) **(A)** Representative colon sections with examples of intact (arrowheads) and degranulated (arrows) MCs. Staining: Toluidine Blue. Sections: 4μm. Scale bars: 20 μm. **(B)** Degranulation of MCs was assessed by an enzymatic assay. Experiments were performed twice with n = 4 animals/group. Values of the percentage of β hexosaminidase release are expressed as the mean ± S.D., n = 8. Data were analyzed using one-way ANOVA (post Tukey multiple-comparison test). **p <0.01 compared to respective WT group. ^##^p <0.01 compared to untreated *in vivo* and *ex vivo* (-/-) and ^&&^p <0.01 compared to treated and not treated *ex vivo* (-/+).

**Figure 3:**
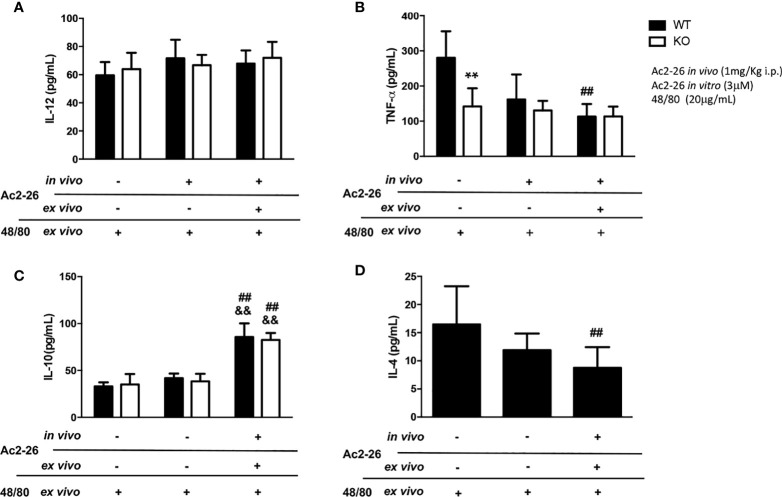
Cytokine production by colon explants from C57BL/6 WT and C57BL/6 IL-4 KO mice treated *in vivo* with 1 mg/kg i.p. Ac_2-26_ (+) or PBS (-). ELISA determined the concentration of IL-12 **(A)**, TNF-α **(B)**, IL-10 **(C)** and IL-4 **(D)** in explants stimulated for 24 h, with 20µg/mL compound 48/80 and with or without 3µM Ac2-26 treatment. Experiments were performed twice with n = 4 animals/group. Values of concentration in pg/mL are expressed as the mean ± S.D, n = 8. Data were analyzed using one-way ANOVA (post Tukey multiple-comparison test). **p <0.01 *vs.* respective WT group. ^##^p <0.01 *vs.* untreated *in vivo* and *ex vivo* (-/-) and ^&&^p <0.01 *vs* treated *in vivo* and not treated *ex vivo* (-/+).

The effects of Ac_2-26_ N-terminal peptide of AnxA1 (Ac-AMVSEFLKQAWFIENEEQEYVQTVK- Invitrogen, USA) were determined by intraperitoneal administration of 1 mg/kg peptide (23). As control, animals received phosphate-buffered saline (PBS). For experiments in **Figures 1****–****3**, 32 male mice were used, being 16 WT and 16 IL4- KO; experiments were performed twice with 4 mice/group. All mice were euthanized for colon extraction. The organ was weighed and measured and, afterwards, divided into four fragments of 1cm for histological analysis and *ex vivo* assays, respectively.

### Histological Analysis

Colonic fragments were opened longitudinally along the entire length and after macro dissection, were fixed in 10% buffered paraformaldehyde for 24 h for histological processing. Fragments were dehydrated in graded ethanol and embedded in paraffin. Serial longitudinal 4µm sections were obtained using microtome (Leica Biosystems, RM2265, Wetzlar, German) and stained with 0.5% Toluidine Blue (Sigma, St. Louis, MO, USA) for MC analysis based on their morphological characteristics. The average number of MC was calculated and recorded. Values are expressed as the mean ± SD of the number of cells per 0.1 mm^2^. Sections were analyzed on an Axioskop 2-mot plus Zeiss microscope (Carl Zeiss, Jena, Germany).

### Explant Culture

Colon fragments were used for explant culture as follows: tissues were transferred to 48-wells plates containing Roswell Park Memorial Institute medium (RPMI, Gibco/Thermo Fischer, Waltham, MA, USA) supplemented with 10% fetal bovine serum (FBS), 1% gentamicin and L-glutamine. Tissues were cultivated with 3µM AnxA1 peptide and 20µg/mL compound 48/80 (Sigma, St. Louis, MO, USA) in a humidified incubator at 5% CO_2_ during 24 h according to (35). After incubation supernatants were collected and 20µg/mL compound 48/80 (Sigma, St. Louis, MO, USA) in RPMI medium was again added to cultures. Then, after 24 h the supernatants collected. The control samples were maintained in supplemented RPMI medium alone. All supernatants were stored at -20°C until cytokine quantification and β-hexosaminidase release assays.

### Quantification of TNF-α, IL-4, IL-10 and IL-12 Cytokines

Cytokines were evaluated using commercial immunoassay kits (Mouse Set OptEIA™ for TNF-α: Code 555268; IL-4: Code 555232; IL-10: Code 555252, BD Biosciences. IL-12: Code 14-7122, BD Biosciences, San Jose, CA, USA), according to the manufacturer’s instructions. For the tests, 25µl of undiluted explant supernatants were used.

### Human MC Line HMC-1

The human MC line HMC-1 was kindly provided by Dr. Joseph H. Butterfield (Mayo Clinic, Rochester, MN, USA) and cultured in Iscove’s Modified Dulbecco’s Medium- (IMDM, Gibco-Thermo Fisher) supplemented with 10% FBS (Cultilab, Campinas, SP, Brazil), 40U/ml penicillin/streptomycin (Sigma) and 1.2 mM α-thioglycerol (Sigma, St. Louis, MO, USA). HMC-1 cells (5×10^5^ cells/well in 24-well plates) were pre-treated with Ac_2-26_ peptide (3, 5 and 10uM) in addition to 5µg/mL of the antagonist of FPRs, BOC-2/Boc-FLFLFL (Sigma, St. Louis, MO, USA) or PBS at 37°C in a humidified incubator at 5% CO_2_. Then, cells were stimulated with 20 µg/mL compound 48/80 (Sigma, St. Louis, MO, USA) at the same conditions. Culture supernatants were collected, and total cell lysates were obtained by adding 0.1% Triton X-100 (Sigma, St. Louis, MO, USA) in PBS to the pellets. The number of viable cells was determined by trypan blue staining.

### β-hexosaminidase Release Assay

The degranulation of MCs was determined by measuring β-hexosaminidase release in the extracellular medium (36). In a 96-well ELISA plate, samples were incubated with 60 µL of the substrate 1mM p-nitrophenyl-N-acetyl-b-D-glycosaminidase in 0.05M citrate, pH 4.5 (Sigma, St. Louis, MO, USA). The plate was incubated at 37°C for 1 h. The reaction was stopped by adding 0.1M Na2CO3-NaHCO3 buffer pH 10. The absorbance was measured in a microplate reader at 415nm. The percentage of β hexosaminidase release was calculated as follows: β-hexosaminidase release rate (%) = 100 × {(supernatant - blank supernatant)/[(supernatant - blank supernatant) + (total cell lysate]}.

### Statistical Analysis

Statistical comparisons were performed with GraphPad Prism 6 (San Diego, CA, USA) using one-way ANOVA followed by Bonferroni multiple comparison post-test or unpaired t-test. All data are the mean ± SD. P values < 0.05 were considered statistically significant.

## Results

### Histological Analysis

To characterize MCs in WT and KO mice, we analyzed and quantified distal colon sections of PBS or peptide Ac_2-26_-treated animals. In both strains we found singly dispersed MCs with no aggregates located predominantly in the lamina propria **(****Figure 1A****)**. The MC density in WT-PBS group was significantly lower compared to peptide Ac_2-26_ treated mice; in the colon mucosa of IL-4 KO mice the frequency of MCs was significantly higher compared with WT PBS group, although for PBS and AnxA1 peptide Ac_2-26_ treated hosts, MC numbers were similar (**Figure 1B**).

### Ac_2-26_ Modulates MC Acute Reaction in Colonic Explants of WT but Not IL-4 KO Mice

Initially, in order to examine the effect of AnxA1 in colonic MCs, C57BL/6 WT and IL-4 KO mice received intraperitoneal administration of peptide Ac_2-26_ or PBS. After 24 h the colon was obtained as described in Materials & Methods for explant cultures under different conditions and the degranulation of MCs was determined by assessing the activity of β-hexosaminidase (**Figures 2A, B**). Resident MCs from the colon of IL-4 KO mice displayed a significantly reduced degranulation induced by compound 48/80 when compared to WT group (p<0.01); on the other hand, the treatment with AnxA1 peptide Ac_2-26_ did not modify the degranulation pattern of MCs in both WT and IL-4 KO mice. However, when explant cultures were re-exposed *ex vivo* to the peptide Ac_2-26,_ the degranulation triggered by compound 48/80 was selectively abolished in WT, but not in IL-4 KO samples (p<0.01).

### Effects of Ac_2-26_ Treatment on the Cytokine Release of Colonic Explants Stimulated by Compound 48/80

In supernatants of colonic explants of WT and IL-4 KO mice stimulated as above we also assessed IL-12, IL-10, TNF- α and IL-4 levels (**Figure 3**). While no differences in IL-12 were observed, after peptide Ac_2-26_ and compound 48/80 stimulation *ex vivo*, we found a significant increment in IL-10 levels, a pivotal regulatory cytokine, in both mouse strains (p<0.01). In contrast to untreated WT group, IL-4 KO mice showed reduced release of the pro-inflammatory cytokine TNF-α, that was not modified by compound 48/80 stimulation *ex vivo* with or without Ac_2-26_ treatment. Interestingly, the treatment with Ac_2-26_ *ex vivo* yielded a significant decrease in TNF-α and IL-4 release upon compound 48/80 stimulation in colonic explants from WT mice (p < 0.05).

### *In Vitro* Ac_2-26_ Peptide Treatment Inhibits Degranulation of HMC-1 Cells

**Figure 4:**
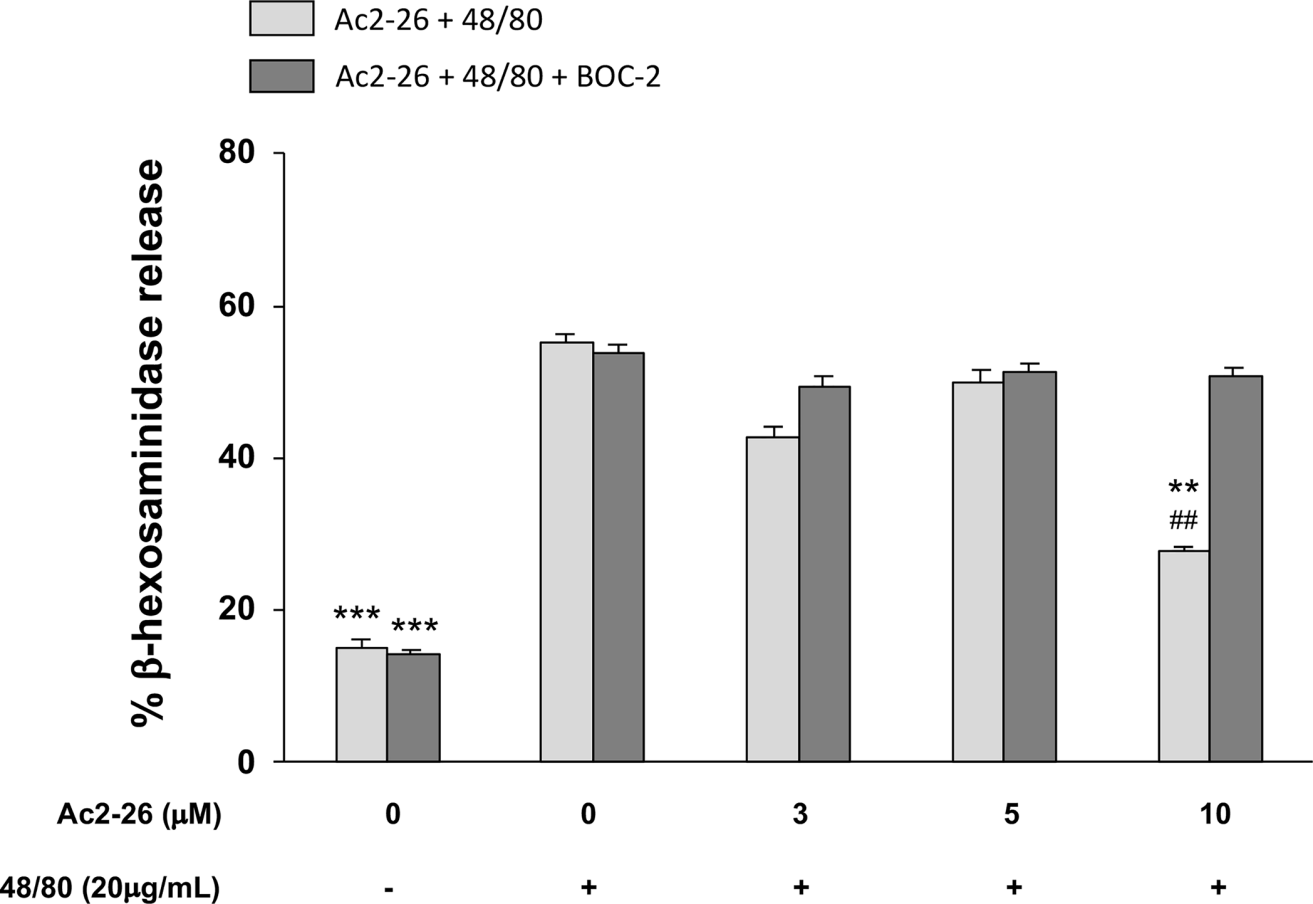
Exogenous AnxA1 (peptide Ac2-26) inhibits HMC-1 cell line degranulation induced by compound 48/80. HMC-1 cells were pre-treated with 3, 5, or 10µM peptide Ac2-26 for 30 min in the absence or presence of FPRs antagonist BOC-2 (5µg/mL). Degranulation was assessed measuring the activity of β-hexosaminidase in culture supernatants. Results are expressed as percentage of enzyme release. All experiments were conducted in triplicate. Data are expressed as the mean ± S.D from four different experiments and analyzed using one-way ANOVA. ***p <0.001 compared to cells treated with 48/80 alone or 48/80 + Ac2-26 (3 or 5 µM); **p <0.01 compared to cells treated with 48/80 + Ac2-26 (10 µM) + BOC-2; ^##^p <0.01 compared to cells treated with 48/80 alone.

**Figure 5:**
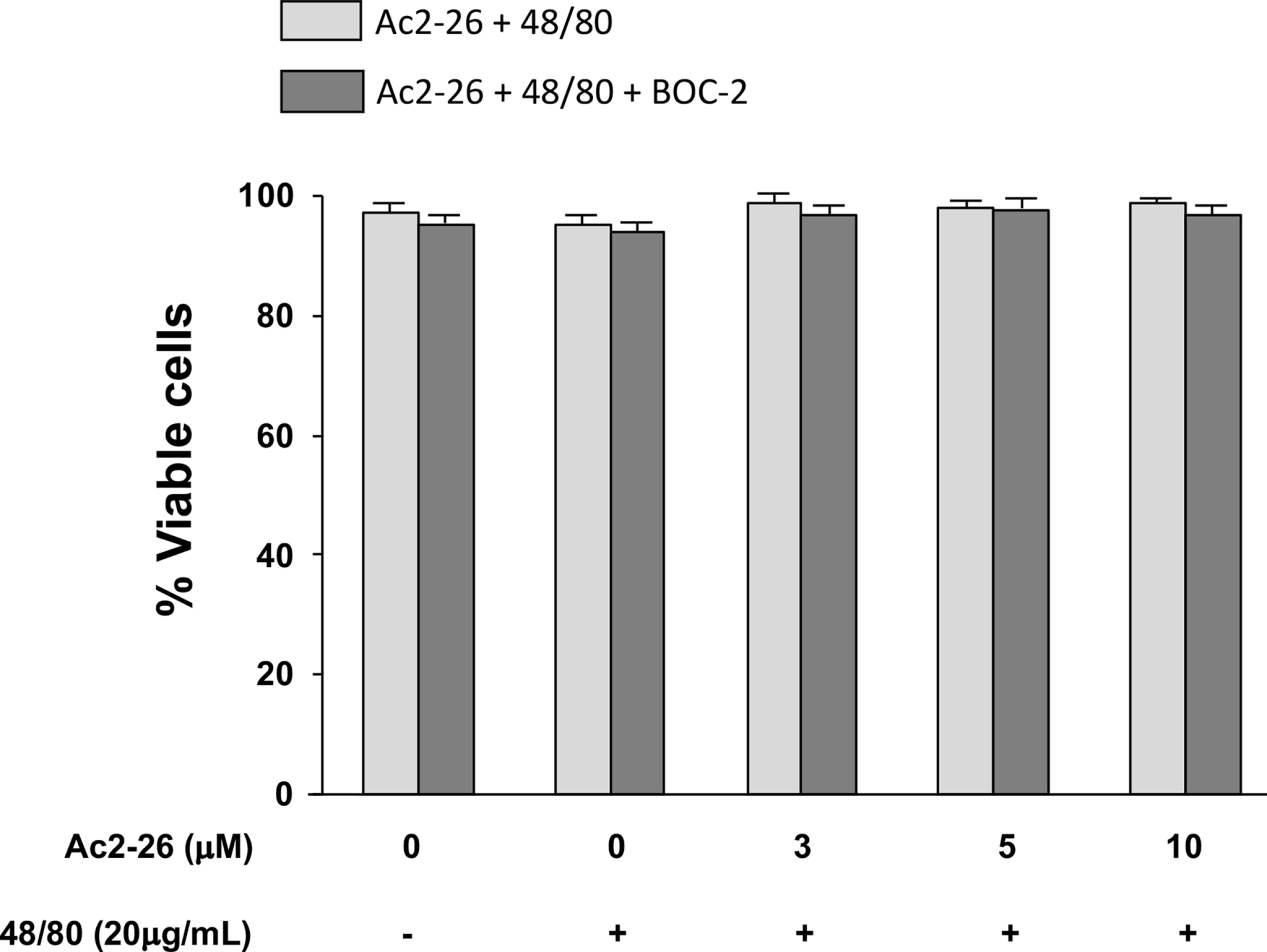
Exogenous AnxA1 (peptide Ac2-26) did not alter viability of HMC-1 cell line. Cells were pre-treated with 3, 5, or 10µM peptide Ac2-26 for 30 min in the absence or presence of FPRs antagonist BOC-2 (5µg/mL). The cells were then stimulated with 20 µg/mL compound 48/80 for 30 min. Cell viability was assessed by trypan blue exclusion assay and results are expressed as percentage. All experiments were performed in triplicate. Data are expressed as the mean ± S.D from four different experiments.

As studies in animals, especially those related to pharmaceutical products, do not always reproduce the complexity of human allergic diseases (37), next we tested the effects of exogenous AnxA1 peptide on acute reaction of human MCs. For that purpose, we took advantage of the well-established cell line HMC-1 to confirm the effect Ac_2-26_. The HMC-1 cells were pre-treated with 3, 5, or 10 peptide Ac_2-26_ (µM), for 30 min prior to stimulation with 20 µg/mL compound 48/80 and the degranulation was assessed by the release of β-hexosaminidase. As shown in **Figure 4**, pretreatment with the optimal dose (10 µM) of peptide Ac_2-26_ significantly inhibited human MC degranulation and this reduction was selectively blocked when FPRs antagonist BOC-2 was added before stimulation. The pre-treatment with both peptide Ac_2-26_ and the antagonist BOC-2 at the doses evaluated did not affect cell viability and survival of HMC-1 cells (**Figure 5**). Collectively, these *in vitro* studies indicate that AnxA1 peptide can inhibit degranulation of HMC-1 cells in a direct pathway, suggesting that the exogenous AnxA1 peptide/FPR axis may alter human MC function.

## Discussion

The MCs are widely distributed throughout most tissues, especially at the mucosal interface where they can respond to a variety of stimuli, with notable differences based on their tissue residency. Previous report using microarray assays indicated that the expression of genes coding for MC proteases Cpa3, Mcpt4, Mcpt5, Mcpt6, and Mcpt10 is restricted to MCs in the peritoneal cavity, which are connective tissue cells. However, the lack of expression of Mcpt1 and Mcpt2 in peritoneal MCs is to be expected, since the expression Mcpt1 and Mcpt2 is found in intestinal MCs (38). Specifically, in the intestine, mucosal MCs express cysteinyl leukotrienes and have high TLR expression, suggesting their commitment to inflammatory responses (39). Due to their location, they secrete a wide range of mediators many of which may affect the intestinal epithelial barrier directly (40).

Depending on the intrinsic secretory phenotype, MCs exhibit different activation profiles from resting, low activation with small secretion of mediators to highly activated (4). In the GI, the MCs exert their biological functions mainly by humoral functions, releasing a range of mediators with some of them involved in monocyte and macrophage activation (TNF-α and IL-6) and others exhibiting regulatory functions (TGFβ1 and IL-10) (41). Remarkably, the perception on MC function has been expanded and now it is well accepted that they perform additional and unexpected activities in strict collaboration with immune and non-immune cells (5). However, little is known about the way MCs interact with or affect the biologic activities of other tissue-resident cells during an inflammatory response.

In this work we found a significantly higher frequency of intact MCs in the colon mucosa of IL-4 KO mice. Consistent with this, IL-4 deficient mice have a higher number of peritoneal MCs (42) and IL-4-deficient bone marrow cell cultures result in a constant increase in the number of MCs and the expression of FcϵRI (43), supporting the role of IL-4 as an endogenous regulator of MC development. On the other hand, after the administration of peptide Ac_2-26_ *in vivo*, while the MC count remained unchanged in the IL-4 KO mice, it increased notably in the WT samples, suggesting an improvement in the identification in the tissue. In agreement, using an intraocular inflammation model, our group demonstrated that the administration of Ac_2-26_ significantly increases the number of the number of intact mast cells and decreased the proportion of degranulated cells (25), confirming that in mice treated with mimetic peptide, its ability to prevent cellular degranulation results in higher numbers of MCs in tissue sections. Furthermore, we found that without any stimulation, the frequency of degranulated MCs in the colon mucosa of WT and IL-4 KO mice was similar, which is in agreement with previous evidence. MC maturation, phenotype and function can vary according to anatomical location, strain background, *in vivo* or *in vitro* studies or cytokine milieu. For instance, IL-4 regulates positively the proliferation and expression of surface receptors, although its deficiency does not influence the number, size, granularity or histamine content, suggesting the compensatory activity of other factors. In the gastrointestinal tract, the largest MC population is found in the mucosa contributing to the regulation of epithelial barrier and participating in both innate and adaptive immune responses. We provide evidence for a negative modulation for basal MC function in the GI tract of IL-4 deficient mice. In agreement, mouse MCs derived from bone marrow cells cultured with IL-3, released higher levels of β-hexosaminidase upon substance P challenge only after 6 days of treatment with SCF plus IL-4 (44). Other groups instead reported that despite a lack of degranulation in response to anti-IgE, peritoneal MCs from IL-4/IL-13- or IL-4R-deficient mice do not have a general degranulation defect, as they responded normally to IgE-independent stimuli such as compound 48/80, or ionomycin (45). One possible explanation for these differences is that the sensitivity of MCs to activation by non-immunological stimuli is dependent on the MC population examined (44). The inability of IL-4 KO mice to orchestrate strong MC activation was not altered by the treatment *in vivo* and *ex vivo* with exogenous peptide Ac_2-26_. However, the IL-4 production in WT mice was affected using the same treatment protocol. Accordingly, in naive T cells from mouse, overexpression of AnxA1 significantly increased IFNγ and reduced IL-4 production, while AnxA1-silenced T cells exhibited decreased IFNγ and increased IL-4 production (46).

It is well accepted that AnxA1 is an important endogenous regulator of MCs, inhibiting their activation when cells are in the resting state and limiting the extent of degranulation and activation response (47). We hypothesize that differences in MC population in normal conditions could determine differential release of MC mediators and the pattern of anaphylactic or acute response after specific stimuli. We uncovered that, after *in vivo* and *ex vivo* administration of Ac_2-26_, WT and KO mice colon explants released higher levels of IL-10 when compared to untreated animals. These results suggest that some of the anti-inflammatory effects of AnxA1 may result from a subsequent release of IL-10 (48). Still, MCs can affect various immune cells by releasing cytokines and chemical mediators (49), therefore the production of IL-10 could be an indirect effect. On the other hand, high expression of TNF-α in activated MCs plays an instrumental role is the pathogenesis of colitis in mice (50). Our data showed an overall reduced production of the pro-inflammatory cytokine TNF-α levels in colon explant cultures from animals lacking IL-4 gene, while administration *ex vivo* of exogenous peptide Ac_2-26_ attenuated TNF-α levels in WT mice. The data highlights that depending on the stimulus, MCs calibrate their pattern of mediator release, modulate the amplification of inflammation, or are involved in the resolution of the immune responses (5). Also, it is difficult to determine the biological relevance of MCs as sources of cytokines in settings where multiple different immune cells represent alternative potential sources of the same products (51). Our findings in the cytokine release experiments illustrate the overall response of the colonic tissue upon compound 48/80 stimulation, which represents a suitable approach to understand how activation or mimetic peptide inhibition of MCs, produces certain outcomes.

Many studies have shown the role of MCs in visceral hypersensitivity reaction mechanisms based on *in vitro* assays using human HMC-1 cell line (52–54). For that reason, the human HMC-1 cell line is considered a good model to evaluate the role of AnxA1 on cell function. Previous reports demonstrated that the release of histamine and prostaglandin D2 by cord-derived human MCs (CBDMCs) activated by IgE-FcRϵ1 crosslinking (55) or treatment with the compound 48/80 (56) was inhibited by pre-treatment with cromoglycate, nedocromil, dexamethasone, and human recombinant AnxA1. Remarkably, our study demonstrated that the AnxA1-derived peptide Ac_2-26_ inhibited *in vitro* the degranulation of HMC-1 cells stimulated with compound 48/80 in FPR-dependent manner, as assessed by blocking the degranulation system using the antagonist BOC-2. Former results with mouse model revealed that AnxA1 is able to downregulate MCs function in allergic disorders (56). Moreover, using human cord-blood derived MCs it has been shown that AnxA1 is an important regulator of MC reactivity to compound 48/80 exerting a negative feedback effect through a mechanism that depends at least partly on the FPR receptor (47).

In summary, Ac_2-26_ showed a strong inhibitory activity in WT colon, reducing the production of TNF-α and IL-4 as well as the MC degranulation. In IL-4 KO mice, the constitutively expanded MCs population seemed less reactive to acute stimulation, although upon Ac_2-26_ treatment, the release of IL-10 the hallmark anti-inflammatory cytokine of the intestinal mucosa-was similar to WT hosts. Herein, we have uncovered properties that include HMC-1 cells with focus particularly on allergic or acute responses. AnxA1 may contribute to biological regulation of human cell line HMC-1, *via* paracrine mechanisms mediated by FPRs. Our study sheds additional light on the function of intestinal MCs and HMC-1 after AnxA1 treatment, delineating new strategies to reduce the release of inflammatory mediators.

## Data Availability Statement

The raw data supporting the conclusions of this article will be made available by the authors, without undue reservation.

## Ethics Statement

The animal study was reviewed and approved by Committee on the Ethics of Animal 145 Experiments from Cordoba National University (resolution 1412/2012).

## Author Contributions

MO designed and performed *in vitro* experiments, helped with the analysis of data and wrote the manuscript. JP performed *in vivo* and *in vitro* experiments. AG performed histological processing and data analysis. SG evaluated the results and contributed to revising the manuscript. SO conceived and coordinated the design of the study, obtained funding for the project, evaluated the results, contributed to drafting the manuscript and supervised the process. All authors contributed to the article and approved the submitted version.

## Funding

This work was supported by the State of Sao Paulo Research Foundation – FAPESP (2019/19949-7), CAPES/MINCyT (99999.000615/2016-01), CEPAM –Unilago and Oswaldo Cruz Institute/Fiocruz.

## Conflict of Interest

The authors declare that the research was conducted in the absence of any commercial or financial relationships that could be construed as a potential conflict of interest.

## Publisher’s Note

All claims expressed in this article are solely those of the authors and do not necessarily represent those of their affiliated organizations, or those of the publisher, the editors and the reviewers. Any product that may be evaluated in this article, or claim that may be made by its manufacturer, is not guaranteed or endorsed by the publisher.
